# Mirizzi Syndrome: From Ultrasound Diagnosis to Surgery—A Case Report

**DOI:** 10.1155/2013/268760

**Published:** 2013-01-10

**Authors:** Dario Pariani, Giorgio Zetti, Fausto Galli, Ferdinando Cortese

**Affiliations:** Azienda Ospedaliera Ospedale di Circolo di Busto Arsizio, Presidio Ospedaliero di Saronno, U.O. Chirurgia Generale e Toracica, Piazzale Borella, 21047 Saronno, Italy

## Abstract

The Mirizzi syndrome is a rare disorder that usually presents with jaundice and cholangitis; its lack of recognition in the diagnostic path could have serious consequences for the patient undergoing cholecystectomy. Here we describe the clinical case of a jaundiced patient from the ultrasound suspect of Mirizzi syndrome to the surgical treatment.

## 1. Introduction

In 1905 Kehr published the first cases of benign extrinsic biliary obstructions caused by gallstones in the gallbladder [1], but it was only in 1948 that Mirizzi reanalyzed and classified this clinical condition, which is characterized by mechanical compression of the common hepatic duct due to a gallstone entrapped into the gallbladder Hartmann pouch or into the cystic duct; therefore from that moment on, this condition was called Mirizzi syndrome [2]. Nowadays the Mirizzi syndrome appears in 1% to 2% of patients with symptomatic cholelithiasis [3] with higher incidence in Central and South America where the reported incidence is 4.7% to 5.7% [3, 4]. Obstructive jaundice and cholangitis are the common presentations of this condition that, if not recognized before operation, makes a cholecystectomy at high risk of damage to the common hepatic duct. Here, we report the case of a patient with Mirizzi syndrome from ultrasound diagnosis to the surgical operation.

## 2. A Case Report

We present the case of a 73-year-old female patient, who arrived in the emergency room for recent onset of epigastric and right hypochondrium pain associated with nausea, vomiting, dark urine, grey feces, and scleroskin jaundice.

Blood tests showed serum total bilirubin 10.23 mg/dL, direct bilirubin 8.62 mg/dL, alanine transaminase (ALT) 190 U/L, aspartate transaminase (AST) 64 U/L, *γ*-glutamyltransferase (GGT) 299 U/L, lactate dehydrogenase (LDH) 334 U/L, and alkaline phosphatase (ALP) 367 U/L.

**Figure 1:**
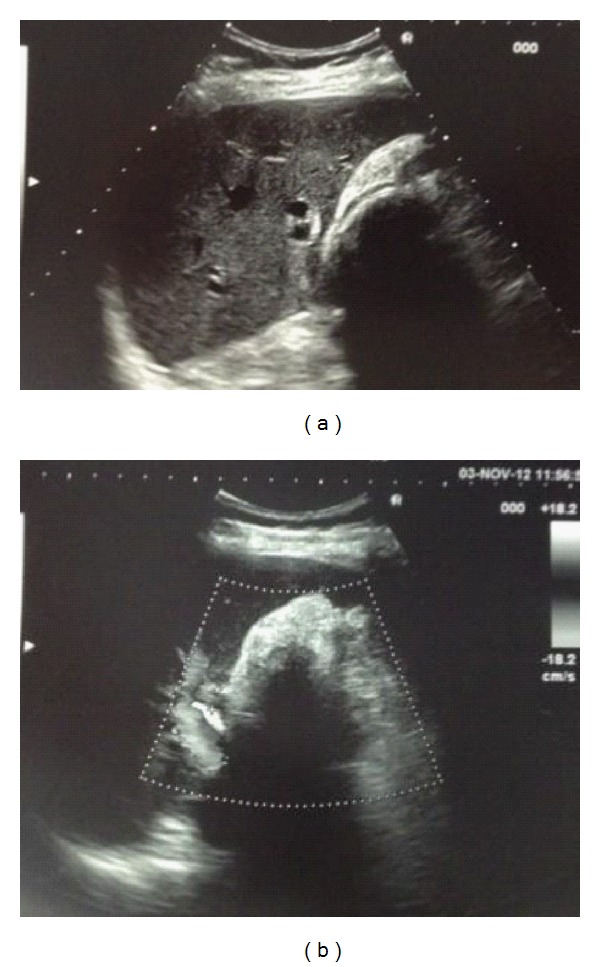
Abdominal ultrasound showing a gallbladder with thick walls and one large gallstone (50 mm) entrapped into the Hartmann pouch.

**Figure 2:**
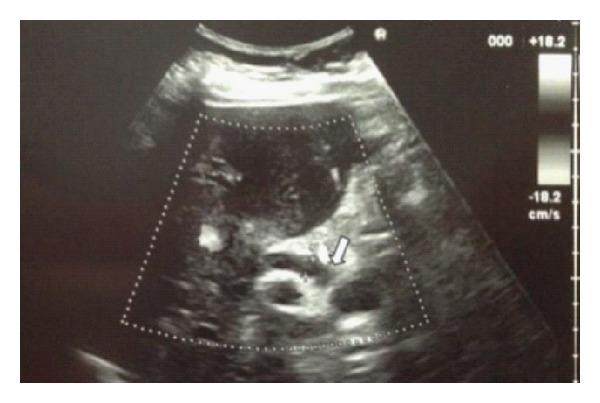
Abdominal ultrasound showing compression of the common hepatic duct which was dilated (8 mm) in its extrahepatic tract above the level of the obstruction (white arrow) and not sonographically observable under the level of obstruction.

Abdominal ultrasound showed a gallbladder with thick walls, one large gallstone (50 mm) entrapped into the Hartmann pouch (Figures 1(a) and 1(b)) and compressing the common hepatic duct which was dilated (8 mm) in its extrahepatic tract above the level of the obstruction and not sonographically observable under the level of obstruction (Figure 2). Intrahepatic biliary ducts were dilated. Neither pancreatic nodules nor Wirsung dilatation wasseen.

**Figure 3:**
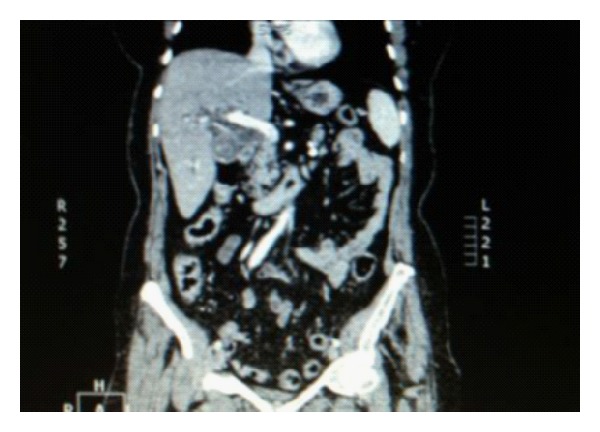
Abdominal computed tomography confirming the ultrasound findings and excluding malignancy in the porta hepatis area, in the liver, and in the pancreas.

An abdominal computed tomography was then performed and confirmed the ultrasound findings, furthermore excluding malignancy in the porta hepatis area, in the liver and in the pancreas (Figure 3).

**Figure 4:**
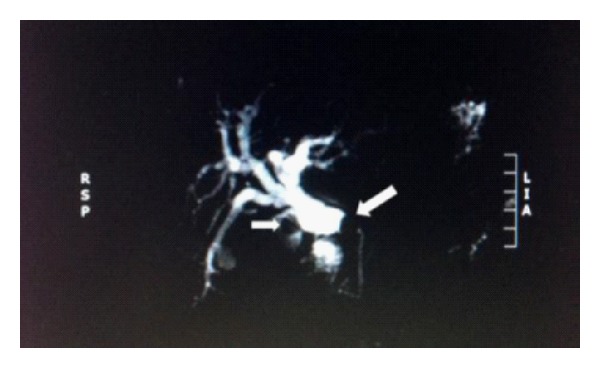
Magnetic resonance cholangiography (MRC) showing presence of one large stone (45 mm) in a gallbladder with thick walls and one smaller stone (12 mm) in the cystic duct with a fistula involving the common bile duct which was dilated above the fistula level and normal below. The white arrows show the two stones.

Magnetic resonance cholangiography (MRC) showed presence of one large stone (45 mm) in a gallbladder with thick walls and one smaller stone (12 mm) in the cystic duct with a fistula involving the common bile duct which was dilated above the fistula level and normal below (Figure 4).

**Figure 5:**
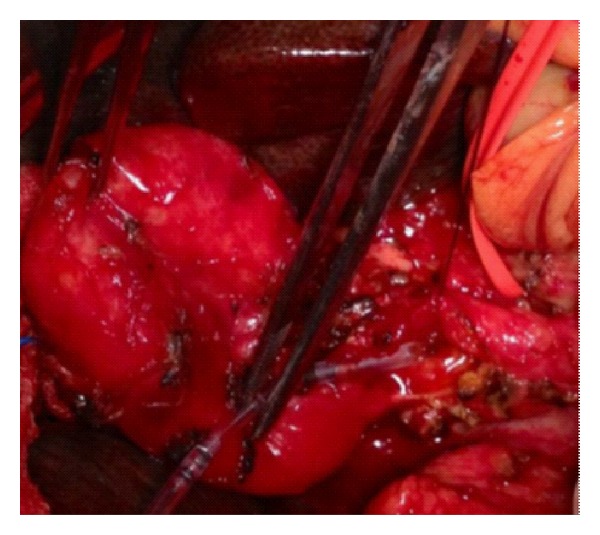
Intraoperative picture showing the common bile duct fistula.

The patient was therefore subjected to surgery. Through a subcostal incision, the gallbladder was detached from the liver, then it was opened, and after removing the bigger stone, the smaller one was seen in the cystic duct with a fistula involving up to two-thirds of the circumference of the common bile duct (Figure 5), hereby the confirmation of a type III Mirizzi syndrome. After removing the smaller stone an operative cholangiogram was performed to confirm the diagnosis and exclude the presence of other stones in the choledocus. 

Through an intraoperative cholangiopancreatography (ERCP) we put a biliary endoprosthesis to cover the fistula, and we also performed a partial cholecystectomy in order to preserve a part of the gallbladder wall to cover the defect in the common bile duct. At the end of the surgical procedure, at the intraoperative ERCP, there was no evidence of any leakages in the biliary tree, and it was seen as a good passage of the contrast medium into the duodenum.

The postoperative course was uneventful with progressive normalization of the hepatic stasis parameters and of transaminases. 

The patient was discharged on the eighteenth postoperative day in good health conditions with no dilatation of biliary tree at the ultrasound control.

## 3. Discussion

Ultrasonography is usually the initial radiological investigation in case of obstructive jaundice. Given the low incidence of the Mirizzi syndrome, an elevated index of suspicion is required to diagnose this condition, and the reported sensitivity of ultrasound in the diagnosis of this disease is 8.3%–27% [5, 6]. 

We always have to suspect the diagnosis of Mirizzi syndrome when, at the ultrasound scans, we see a contracted gallbladder with thick or extremely thin walls with one large gallstone or multiple smaller gallstones being entrapped into the Hartmann pouch or into the cystic duct; furthermore the hepatic duct would be dilated in its extra and intrahepatic tracts above the level of the obstruction site, and the common bile duct would be within normal size or not sonographically observable under the level of obstruction [7].

When at the ultrasound study the suspect of Mirizzi syndrome is high; it is always indicated to subject the patient to a computed tomography which has a higher sensitivity (42%) than the ultrasound [8], and it is also important in order to exclude malignancy in the porta hepatis area or in the liver, even if the presence of periductal inflammation can be misinterpreted as gallbladder cancer [9].

Magnetic resonance cholangiography study is important to clarify whether a fistula is present or not and to exclude choledocholithiasis or other causes of bile tract obstruction. However, the diagnostic accuracy for magnetic resonance cholangiography in the diagnosis of Mirizzi syndrome is 50% [10].

The highest degree of sensitivity in the diagnosis of Mirizzi syndrome reported in the literature is that of endoscopic retrograde cholangiopancreatography (ERCP), whose accuracy is 63% [8]. ERCP is important not only for diagnosis but also as part of the treatment of some cases of Mirizzi syndrome.

In 1989 Csendes et al. published a new classification of patients with Mirizzi syndrome and cholecystobiliary fistula. Type I lesions: external compression of the common bile duct; type II lesions: presence of a cholecystobiliary fistula with erosion of less than one-third of the circumference of the bile duct; type III lesions: the fistula involves up to two-thirds of the duct circumference; type IV lesions: complete destruction of the bile duct [11]. Recently, a new type of fistula was added within this classification system: a cholecystoenteric fistula that complicates the other types of cholecystobiliary fistulae [12].

According to this classification, different surgical strategies are used to treat Mirizzi syndrome: if the fistula is small and eroded less than one-third of the circumference of the common bile duct, the defect can be sutured with fine absorbable sutures and a T tube can be placed distal to the fistula for 1 or 2 months. If the defect is larger, a cuff of the gallbladder is used for fistula closure and a T tube is placed distally. In some patients who present with stricture of the common bile duct due to this fistula, immediate hepaticojejunostomy may produce good long-term results. In type IV lesions it is suggested to perform immediate bilioenteric anastomosis or hepaticostomy leaving a stent or T tube in place for a long time [13]. Despite the fact that some studies suggest the use of laparoscopic surgery to treat Mirizzi syndrome, this approach cannot currently be recommended as a standard procedure because of the increased risk of bile duct injuries [14].

## 4. Conclusion

Our reported case stresses the importance of diagnostic ultrasound in the jaundiced patients. Each sonographer should know about the existence of Mirizzi syndrome, and, in case of ultrasound suspect, he should refer the patient to the best diagnostic and therapeutic path. As regards the use of biliary endoprosthesis, we believe that it could be a valid and better tolerated alternative to the T tube in some cases of biliary repair.
